# Editorial: Canine melanoma in comparative oncology: Translate research advances to develop new diagnostic and therapeutic options

**DOI:** 10.3389/fvets.2022.1127527

**Published:** 2023-01-09

**Authors:** Laura Bongiovanni, Chiara Brachelente, Steven Dow, Philip J. Bergman

**Affiliations:** ^1^Department of Veterinary Medicine, University of Teramo, Teramo, Italy; ^2^Department of Biomolecular Health Sciences, Faculty of Veterinary Medicine, Utrecht University, Utrecht, Netherlands; ^3^Department of Veterinary Medicine, University of Perugia, Perugia, Italy; ^4^Flint Animal Cancer Center, Department of Clinical Sciences, College of Veterinary Medicine and Biomedical Sciences, Colorado State University, Ft. Collins, CO, United States; ^5^VCA Clinical Studies, Los Angeles, CA, United States

**Keywords:** dog, melanoma, translational research, comparative oncology, diagnostics, therapy, biomarker, immunotherapy

Canine melanoma is one of the most studied tumor of the dog, due to its high aggressiveness, especially in its mucosal (oral) form, but also because its similarity with human melanoma. Canine and human mucosal melanoma show several common characteristics, in terms of histological features, biological behavior and genetic modifications (1–3). In both species, malignant melanoma (MM) is a highly aggressive tumor of skin and mucosae, often associated with aggressive malignant behavior with a rapid invasion of surrounding normal tissues, frequent metastasis and resistance to therapy. Despite numerous advances in diagnostic and available treatment options, MM remains fatal in most cases. For this reason, there is currently an urgent need to better understand genetic and molecular mechanisms driving melanoma development and progression, to find valuable new prognostic biomarkers, and novel, effective therapeutic approaches to improve patient survival rates. The aim of this Research Topic was to provide an overview of the current advances in the diagnostic and treatment approaches for canine melanoma in comparative oncology, in light of the translating potential of most of these studies to human melanoma patients (Figure 1). Indeed, most of the research papers published on this Research Topic aimed to improve current diagnostic approaches and treatments for dogs affected by malignant melanoma, but also provided the potential of translating these results to human MM, in particular the rare form of human mucosal melanoma. The present Research Topic is made of 12 original articles of various types, with the majority being original research articles, submitted by the most prominent research groups currently working on canine melanoma all over the world. The great participation we found clearly indicates the importance of this Research Topic in the veterinary research community, mirroring the importance of the disease in the daily clinical work of both dogs and men.

**Figure 1:**
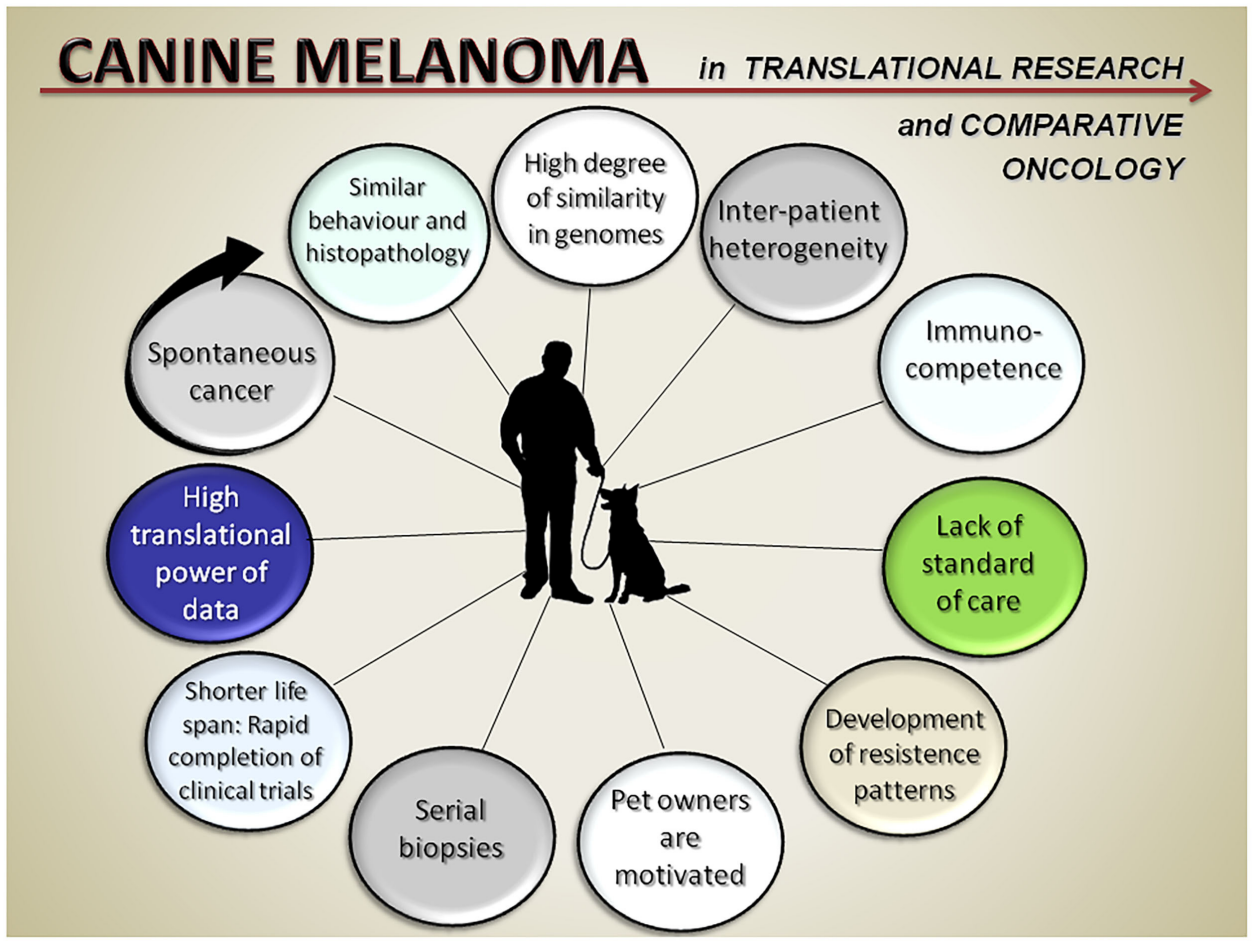
Points of strengths of the canine melanoma spontaneous model in translational research and comparative oncology.

We include in the following paragraphs a brief summary of the main findings reported by different authors in their 12 manuscripts that form the bulk of this Research Topic.

A perspective article is included in the present Research Topic, where authors underlined the relevance of translational research performed in last years on canine melanoma. In the paper, the most relevant knowledge on the tumor landscape of canine melanoma is reported. Furthermore, starting on the observation of the great achievements by immunotherapy in the treatment of human melanoma patients, the most promising immunotherapeutic approaches recently investigated, such as anti-cancer vaccination, are reviewed and critically discussed in the paper of Tarone et al.. A brief introduction on the importance of extracellular vesicles as valuable circulating carriers of easily accessible cancer biomarkers is also included, to discuss the potential of these novel approaches to improve the diagnosis and prognosis of canine MM.

The current status of canine melanoma treatment and diagnostics in Brazil, reported from a “Colloquium on Canine Melanoma,” a meeting organized by the Brazilian Association of Veterinary Oncology (ABROVET) on December 2020, has been summarized in an opinion paper (Fonseca-Alves et al.). The success of this meeting, with more than 100 attendees, testifies the attention and interest on this Research Topic of the Brazilian oncology community.

A total of six articles that deal with the evaluation of new diagnostic and prognostic markers for canine MM have been published. Some of them have been already reviewed and included in the recently published review paper “*Diagnosis and Histopathologic Prognostication of Canine Melanocytic Neoplasms: A Consensus of the Oncology-Pathology Working Group*” (4). Global DNA methylation has been investigated and quantified in canine melanotic and amelanotic oral MM samples and in the peripheral blood leukocytes as a novel biomarker, presuming a predominant global DNA hypomethylation in these tumors, potentially related to Ki67 score and tumor pigmentation (Scattone et al.). In their study, Tsoi et al. investigated the IHC and RNA expression patterns of a set of additional markers, besides the well-known melanocyte markers Melan A, PNL2 and TRP-1 and−2, to investigate their potential usefulness for the challenging differentiation of spindloid oral MMs from soft tissue sarcomas (STS), when junctional activity and pigmentation are absent. Authors found that TYR, CD34, and CALD1 were the most discriminatory genes in differentiating between oral MM and STS, and should be evaluated in suspected amelanotic OMMs. The gene expression of H2AFZ and survivin in formalin-fixed, paraffin-embedded canine melanoma samples has been related to malignant histological features and malignant behavior, indicating H2AFZ as a new potentially useful prognostic biomarker and confirming survivin as a useful prognostic biomarker when evaluated also at the RNA levels (Bongiovanni et al.). Leukotriene A4 hydrolase (LTA4H), and Fragile-X-mental retardation-related protein1 (FXR1), both reported as related to metastatic potential in different tumors, appeared to be highly expressed in oral melanoma, at both protein and mRNA level, but did not correlate to known histologic and immunohistochemical prognostic markers. However, their extensive expression in the investigated cases suggests that they may play a role in canine oral melanoma oncogenesis (Nordio et al.). Silveira et al. demonstrated COX-2 overexpression in both oral and cutaneous melanomas and observed that COX-2 expression correlates with established markers of poor prognosis. Porcellato et al. investigated the presence and significance of tumor-associated macrophages in canine melanocytic tumors, through the use of several IHC biomarkers (CD163, CD204, Iba1, and MAC387), revealing an association of these markers with negative prognostic histologic features and a more aggressive biological behavior.

Studies on experimental therapeutic approaches exploiting immune-, gene- or targeted therapy were received and included in this Research Topic, showing interesting and encouraging results.

More than one group of researchers is currently engaged in the study and discovery of new targets for the development of new therapeutic approaches by the use of *in vitro* models of canine melanoma. Silveira et al., by silencing Cox-2 in two melanoma cell lines, demonstrated that cellular proliferation, migration and invasion are COX-2 dependent, establishing that COX-2 is an essential driver of cellular proliferation, migration and invasion, and its expression correlated with malignant behavior in canine melanoma. Alpha-connexin carboxyl-terminal peptide (aCT1) and Bowman-birk protease inhibitor (BBI) treatment, alone or in combination, targeting Cx43, were investigated by Sato et al. on canine oral melanoma cell viability. Authors found that this dual treatment can be combined to achieve anticancer activity. The possibility of blocking the Rb-E2F pathway by using a CDK4/6 inhibitor (Palbociclib) was investigated by Bongiovanni et al. as a potential anti-cancer therapy on four different canine oral melanoma cell lines. The selected drug was effective on three of the four tested cell lines, including a metastatic melanoma-derived one, indicating that CDK4/6 inhibitors could potentially be used as a new anti-cancer treatment for canine melanoma.

The immunologic components of the immune environment and the mechanisms of evasion of the immune response by melanoma cells have been investigated in several papers on this Research Topic. The different T cell phenotypes and functions in melanoma tumor tissue and PBMCs of melanoma-affected patients were evaluated by Sparger et al., revealing a different and unique T cell phenotype of both unstimulated and stimulated T cell populations in melanoma patients compared to healthy dogs. Takeuchi et al. demonstrated that the immunosuppressive cytokine transforming growth factor beta 1 (TGF-β1) is upregulated *in vitro* and *in vivo* in oral malignant melanoma and that its suppressive effect could be reverted *in vitro* by a decoy receptor, developed by the authors, exploring the potential application of this receptor in biologic therapy.

Included in the present Research Topic is also a study investigating the approach of gene therapy using adenoviruses encoding the immunomodulatory gene CD40L (AdCD40L). This strategy has already shown promise in clinical trials on human melanoma patients. Saellström et al. reported results on local AdCD40L treatment in dogs with different types of melanoma (mainly oral and cutaneous), indicating that this type of therapy is safe and could have beneficial effects in canine patients and potential valuable clinical translation to human patients.

Altogether, these manuscripts present new insights on the current researches on canine melanoma with the specific aim to improve our current diagnostic and therapeutic approaches to this highly aggressive and lethal disease of the dog, with a high translational potential to human mucosal melanoma.

## Author contributions

All authors listed have made a substantial, direct, and intellectual contribution to the work and approved it for publication.
